# Supplementary figures and images for: Endoplasmic reticulum retention motif fused to recombinant anti-cancer monoclonal antibody (mAb) CO17-1A affects mAb expression and plant stress response
Source: PLoS One. 2018 Sep 24;13(9):e0198978. doi: 10.1371/journal.pone.0198978 (PMC6152870; doi:10.1371/journal.pone.0198978)

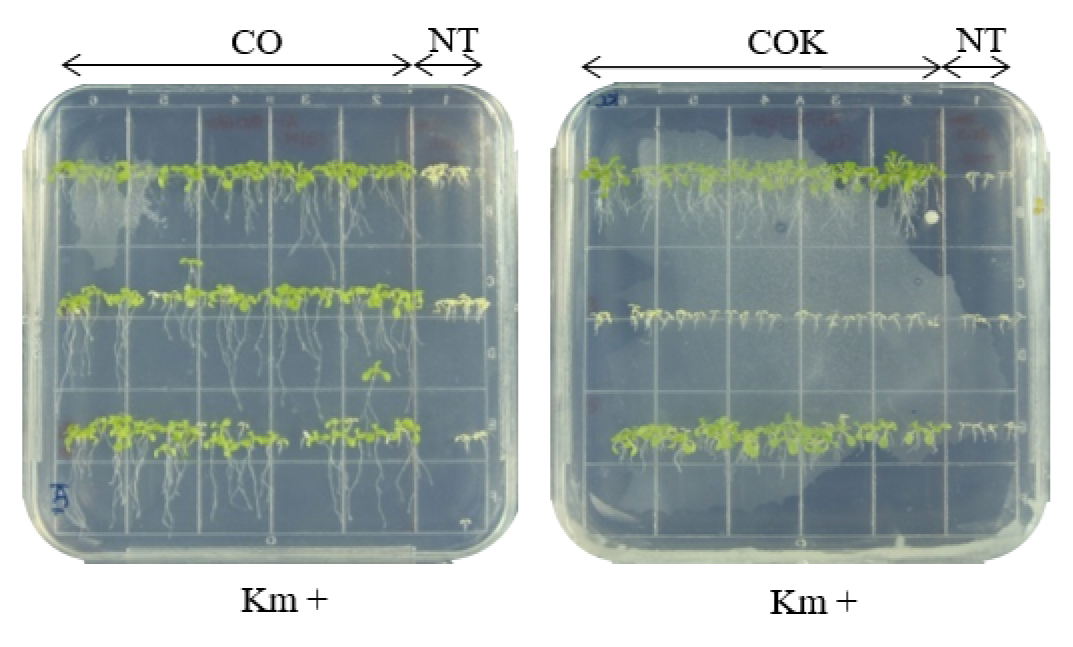

Supplement: S1 Fig — Kanamycin selection was repeated over generations to obtain homozygous seeds for further study. Photographs were taken 14 days after seed germination using a camera (Digital Gross System) (Humintec, Suwon, Korea). CO: seedling expressing mAbP CO; COK: seedling expressing mAbP COK; Km+: kanamycin containing media. (TIF) [file pone.0198978.s001.tif]

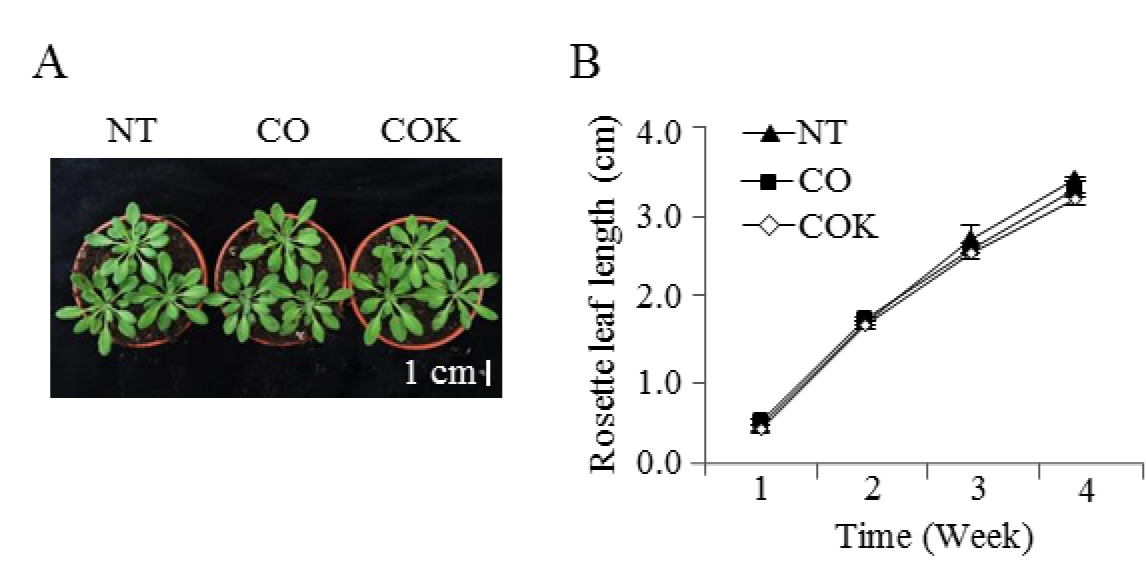

Supplement: S2 Fig — (A) Seeds (NT, mAb CO, and mAb COK) were sown into pots containing soil in a growth chamber. Plants were photographed one month after transfer to pots. NT: non-transgenic plants; CO: transgenic plants expressing mAb CO; COK: transgenic plants expressing mAb COK. (B) Rosette leaf lengths were measured from the petiole to the blade using a ruler at 1-week intervals after transplantation from in vitro conditions. Scale bar represents 1 cm in each photograph. (TIF) [file pone.0198978.s002.tif]
